# Composition Design Strategy for High Entropy Amorphous Alloys

**DOI:** 10.3390/ma17020453

**Published:** 2024-01-18

**Authors:** Hongyu Ding, Qi Zhang, Kefu Yao

**Affiliations:** 1 Marine Equipment and Technology Institute, Jiangsu University of Science and Technology, Zhenjiang 212003, China; 2 School of Materials Science and Engineering, Tsinghua University, Beijing 100084, China; 3 School of Materials Science and Engineering, Jiangsu University of Science and Technology, Zhenjiang 212003, China; 231110601229@stu.just.edu.cn

**Keywords:** high entropy alloy, amorphous alloy, bulk metallic glass, glass-forming ability, composition design strategy

## Abstract

High entropy amorphous alloys (HEAAs) are materials that have received much attention in recent years. They exhibit many unique properties; however, research on their composition design method has not been deep enough. In this paper, we summarized some effective composition design strategies for HEAAs. By adjusting the atomic ratio from quinary bulk metallic glasses, Ti_20_Zr_20_Cu_20_Ni_20_Be_20_ HEAA with a high fracture strength of 2315 MPa was designed. By similar element addition/substitution, a series of Ti–(Zr, Hf, Nb)–Cu–Ni–Be HEAAs was developed. They possess good glass-forming ability with a maximum critical diameter of 30 mm. Combining elements from those ternary/quaternary bulk metallic glasses has also proved to be an effective method for designing new HEAAs. The effect of high entropy on the property of the alloy, possible composition design methods, and potential applications were also discussed. This paper may provide helpful inspiration for future development of HEAAs.

## 1. Introduction

Material innovation has become one of the most important driving forces for promoting human civilization progress, as well as promoting the development of technology and industrial upgrading. Amorphous alloys and high entropy alloys are two types of high-performance materials that have developed rapidly in the past several decades. Since its first report in 1960 [1], amorphous alloys have undergone significant development and have now expanded to dozens of material systems. Inoue et al. developed a copper mold casting method that greatly reduced fabrication costs [2,3]. Peker and Johnson designed a very famous amorphous alloy, Zr_41.2_Ti_13.8_Cu_12.5_Ni_10_Be_22.5_, which was also called Vit1 since it possesses very good glass-forming ability (GFA) [4]. Vit1 played an important role in promoting the industrialization of amorphous alloys. Inoue presented a long review of amorphous alloys, and it got more than 5600 citations [5]. After entering the 21st century, more and more amorphous alloys were developed, such as Cu–Zr–Ti–Sn [6], Ni–Nb–Sn [7], Pt–Co–Ni–Cu–P [8], Zr–Al–Co [9], Zr–Cu–Al [10], Cu–Zr–Ag [11], etc.

One of the main challenges in developing amorphous alloys is how to improve its GFA. Li et al. found that similar atom substitution may be an effective way [12,13]. Santos et al. proposed a topological instability (λ) criterion to evaluate GFA in an Ni–Nb–Zr system [14]. Zhang et al. developed the Ti_32.8_Zr_30.2_Ni_5.3_Cu_9_Be_22.7_ quinary bulk amorphous alloy; it possesses good GFA and its critical diameter exceeds 50 mm [15]. Nishiyama et al. prepared the world’s biggest glassy alloy, namely the Pd_42.5_Cu_30_Ni_7.5_P_20_ cylindrical glassy alloy sample. Its diameter was 80 mm and it was obtained by fluxing and water quenching method [16]. Apart from experiments, several parameters were proposed for evaluating the GFA of an alloy, such as *T*_rg_ [17], Δ*T*_x_ [18], *γ* [19], etc. The atomic simulation method was also applied to enhance the GFA of Ni–Nb–Ti amorphous alloys by Li et al. [20]. Amorphous alloys possess outstanding performance, such as high strength, high hardness, good corrosion resistance, and wear resistance, etc. As a result, amorphous alloys are used as low-loss power transformers, precise forming parts, micro-electro-mechanical system components, etc.

The concept of a high entropy alloy (HEA) has received widespread attention from the material research community since its first report in 2004 [21]. High entropy alloys usually contain five or more elements while the concentration of each element was in the range between 5% and 35%. In other words, the configurational entropy of high entropy alloy should be greater than 1.5 *R*, in which *R* represents the ideal gas constant (8.314 J/(mol·K)). For alloys containing five elements with equal atomic concentration, the configurational entropy reaches 1.61 *R*. In this sense, they are called high entropy alloys. In thermodynamics, entropy is a parameter characterizing the degree of disorder in a system. The degree of disorder increases as the number of constituent elements increases. Unlike traditional alloys that are based on one or two principle elements with other elements as minor additions, high entropy alloys belong to multicomponent, non-principle element alloy systems. Due to its breakthrough in traditional alloy design concepts, a new door for material research has opened up, making thousands of material combinations possible. As indicated by calculation, an array choice including 13 mutually miscible metallic elements enables 7099 high entropy alloy systems with 5 to 13 elements in equal molar ratios [21]. It provides a wide range of space and possibilities for developing new alloys.

Cantor et al. developed a series of multicomponent alloys. It was found that the total number of phases is always well below the maximum equilibrium number allowed by the Gibbs phase rule. Among them, a Fe_20_Cr_20_Mn_20_Ni_20_Co_20_ alloy possesses an FCC structure [22]. This alloy was called “Cantor alloy”, and it was intensively studied by other researchers. For example, Gludovatz et al. found that the Cantor alloy possesses exceptional damage tolerance with tensile strengths above 1 GPa and fracture toughness values exceeding 200 MPa·m^1/2^; it is fracture resistant for cryogenic application [23]. Shahmir et al. provided an overview on microstructural engineering of the Cantor alloy in the past twenty years [24]. Zhang et al. proposed that phase formation of HEA can be separated by mixing enthalpy Δ*H*_mix_ and atomic-size difference *δ*, it provides important guidance in designing HEAs with desired phases and microstructure [25]. Yamabe-Mitarai et al. studied the stability of Ti-containing high-entropy alloys, it was found that strengths of the BCC HEAs were greater than those of the HCP HEAs at 873 K, they were also greater than that of the commercial Ti alloy TIMETAL 834, indicating that BCC HEAs may be applied at elevated temperatures [26]. Uporov et al. found that ScGdTbDyHo HEA possesses good magnetocaloric properties and it can be influenced by the synthesis route [27]. Most HEAs were prepared by casting method; recently it was found that additive manufacturing characterized by net-shape processing is suitable for elevating the properties of HEAs [28,29,30]. Overall, great progress has been made in phase forming rules, composition design, processing, and application of HEA under various circumstances; they may be potential materials applied in many fields, such as heat-resistant and wear-resistant coatings, magnetic materials, and extreme high/low-temperature materials, etc.

Many factors could affect the phase formation of HEAs. In most cases, HEAs form solid solutions (especially BCC, FCC, and HCP) or intermetallics. However, under certain conditions, an amorphous structure could also be formed; this is high entropy amorphous alloy (HEAA). HEAAs possess both the long-range disordered atomic structure stacking characteristics of amorphous alloys and compositional complex characteristics in high entropy alloys. They are a new type of multiple component-disordered alloy. In other words, HEAA comprises five or more elements with an atomic ratio of each element between 5% and 35%, while it possesses an amorphous structure at room temperature. From a scientific research perspective, HEAAs provide a model material connecting amorphous alloys and high entropy alloys; it is helpful for intensive research on the amorphous forming rule of amorphous alloys and the phase evolution mechanism of high entropy alloys. From an industrial application perspective, due to complicated composition and structural characteristics, HEAAs exhibit a series of unique physical, chemical, and mechanical properties; they may be applied on certain specific occasions.

So far, positive progress has been achieved and dozens of HEAAs have been developed. The first batch of HEAAs was reported in 2002 by Ma et al., namely Ti_20_Zr_20_Hf_20_Cu_20_M_20_ (M = Fe, Co, Ni) alloys. Among them, the Ti_20_Zr_20_Hf_20_Cu_20_Ni_20_ alloy can form bulk metallic glass (BMG) with a critical diameter of 1.5 mm. They were called multicomponent glassy alloys or non-base glassy alloys at that time [31]. Later in 2011, the research work continued. Zhao et al. prepared a Zn_20_Ca_20_Sr_20_Yb_20_(Li_0.55_Mg_0.45_)_20_ BMG and it possesses homogeneous flow behavior at room temperature [32]. Takeuchi et al. developed the Pd_20_Pt_20_Cu_20_Ni_20_P_20_ alloy, its critical diameter reaches 10 mm and the concept of high entropy bulk metallic glass (HE-BMG) was proposed [33]. Li et al. developed CaMgZnSrYb HE-BMG with good biodegradable properties [34]. Later, Yao’s group developed a series of Ti–Zr–(Hf)–Cu–Ni–Be HE-BMGs with good GFA [35,36,37,38]; the composition design method will be discussed in detail later. Kim et al. developed Er–Gd–Y–Al–Co HE-BMGs and found that the relation between the fragility and elastic properties of these alloys is quite different from traditional BMGs [39]. Xu et al. developed Fe_25_Co_25_Ni_25_ (P, C, B, Si)_25_ HE-BMGs with good magnetic properties [40]. Huo et al. developed a denary HE-BMG with a large magnetocaloric effect [41]. Bizhanova et al. developed quinary Zr_31_Ti_27_Be_26_Cu_10_M_6_ (M = Ag, Al, Ni, V, Cr, and Fe) and senary Zr_28_Ti_24_Be_23_Cu_9_Ni_10_N_6_ (N = V, Cr, Fe, Ag, and Al) alloys with critical diameters of 6–15 mm [42]. Inoue et al. found that Fe_43_Cr_16_Mo_16_C_15_B_10_ HE-BMG and Zr–Al–(TM1, TM2) pseudo-HE-BMG can confer useful heat resistance at elevated temperatures [43]. Wada et al. developed septenary Zr–Hf–Ti–Al–Co–Ni–Cu high-entropy bulk metallic glasses with centimeter-scale glass-forming ability [44]. Panahi et al. studied the glass forming range of (FeCoCrNi)–(B,Si) HEAAs, the crystallization process, and the influence of Si element on the microstructure was elucidated [45]. Szyba et al. studied structural and electrochemical properties of resorbable Ca_32_Mg_12_Zn_38_Yb_18–x_B_x_ (x = 1, 2, 3) metallic glasses in Ringer’s solution; it was found that the HEAA had significantly higher corrosion resistance than CaMgZn alloys [46]. Law et al. compared the magnetocaloric properties of amorphous and crystalline HEAs; it was found that the magneto-entropy change of HEAAs was generally larger than its crystalline counterpart, while the transition temperature was relatively lower [47]. Calin et al. found that Ti–Zr–Nb–Hf–Si HEAAs exhibit excellent corrosion properties in simulated body fluids. Moreover, its weak paramagnetic nature and superior radiopacity offer compatibility with medical diagnostic imaging systems [48]. Jalali et al. studied the thermal and deformation behavior of Zr_33_Hf_8_Ti_6_Cu_32_Ni_10_Co_5_Al_6_ HE-BMG; the correlation between fragility, structural relaxation enthalpy, diffusion, free volume and deformation behavior was discussed compared with the Cu–Zr–Al prototype BMG [49,50]. Jia et al. created a nanosponge-like architecture from PdPtCuNiP HEAA; it possesses outstanding hydrogen evolution reaction activity [51]. Makarov et al. studied temperature dependencies of enthalpy change in the initial (as-quenched) and relaxed (aged) HE-BMGs; the calculated results agreed with interstitialcy theory [52]. Alvi et al. reported that a thin film of HfMoNbTaTiVWZr HEAA showed thermal stability up to 750 °C, and it can resist Ar-ion irradiation [53]. Ding et al. developed HE-BMG by similar element substitution/addition [54]. Moreover, quinary HEAAs can also be designed from the existing three kinds of ternary BMGs [55]. Cemin et al. designed NbTaTiVZr(O) HEAA coating by magnetron sputtering deposition; the surface was completely passivated. Moreover, corrosion resistance and hydrophilicity were also increased compared with crystalline samples [56]. Ohashi et al. designed a new Zr_35_Hf_13_Al_11_Ag_8_Ni_8_Cu_25_ HE-BMG based on a high-entropy strategy, and its critical diameter reaches 20 mm [57]. Li et al. prepared a TiNiSiCrCoAl high-entropy alloy coating on the Ti-6Al-4V surface; the matrix phase was an amorphous structure, and the σ phase with an FCC structure precipitated. The coating possesses good oxidation resistance at high temperatures [58]. Hussain et al. welded Cu–Hf–Ni–Ti–Zr HE-BMG and Ti-22Al-27Nb alloys together to improve the tensile ductility of the latter alloy [59]. Ding prepared Ti–Zr–Cu–Ni–Al–Co HEAA/nanocrystalline coating on Ti-6Al-4V surface to improve its wear resistance [60]. Ding et al. suggested that combining elements from existing quartenary BMGs can also be an effective way of designing quinary HEAAs [61]. Bazlov et al. found that the replacement of Mo by V in Fe–Co–Ni–Cr–(Mo, V)–B HEA leads to thermal stability enhancement of the amorphous phase [62]. These works established foundations for subsequent research on HEAAs.

In general, the HEAA family is still very small in thousands of high entropy alloys, as well as in amorphous alloys. Most high entropy alloys do not form amorphous phases. Meanwhile, lots of amorphous alloys contain three to 4fourelements instead of more than five elements. The comprehensive theory/method for high entropy amorphous alloy design was rarely seen. The technical difficulty in designing the composition of HEAAs lies in the type and proportion of elements; there is a high probability that the designed high entropy alloy may not obtain an amorphous structure by randomly choosing a combination of elements. At present, research on the composition design strategy of HEAAs has not been reported or discussed in-depth enough. This review attempts to summarize some effective methods and strategies for the composition design of HEAAs (including HE-BMGs), discuss the effect of high entropy on the property of the alloy, possible composition design methods, and potential applications in the future. This work would be beneficial for promoting the development and applications of HEAAs.

## 2. Composition Design of HEAAs

### 2.1. Designing HEAA Based on Quinary Bulk Metallic Glasses

It is well known that the key point for preparing amorphous alloys is avoiding crystallization of high-temperature alloy melt during the cooling process. When an amorphous alloy could be obtained at a low cooling rate, or the critical size for obtaining an amorphous metallic sample is large, the alloy is recognized as possessing good or large glass-forming ability (GFA). Among traditional amorphous alloys, Zr_41.2_Ti_13.8_Cu_12.5_Ni_10_Be_22.5_ (Vit1) [4] and Ti_32.8_Zr_30.2_Ni_5.3_Cu_9_Be_22.7_ [15] quinary bulk amorphous alloys possess good GFA and a critical diameter over 50 mm. It implies that the five elements, Ti, Zr, Cu, Ni, and Be, are structural and chemically compatible to form bulk metallic glasses. So it is reasonable to suppose that the Ti_20_Zr_20_Cu_20_Ni_20_Be_20_ high entropy alloy may possess good glass-forming ability and a big glassy sample might be made.

**Figure 1:**
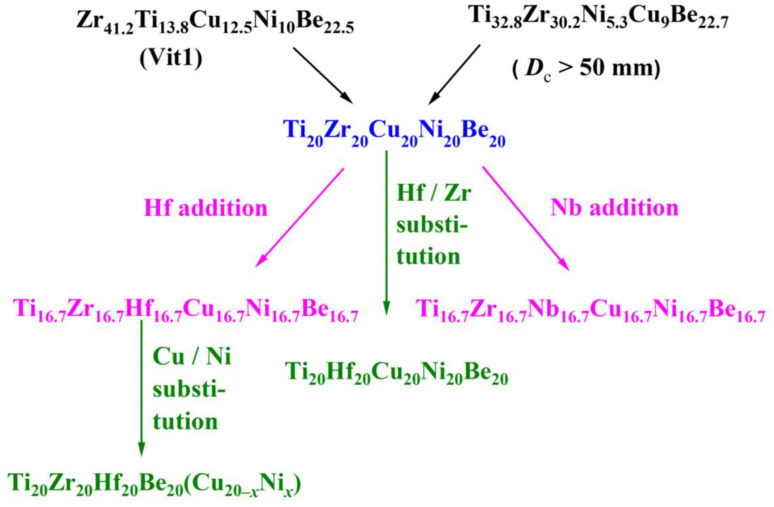
Composition design approach for Ti–(Zr, Hf, Nb)–Cu–Ni–Be HEAAs.

**Figure 2:**
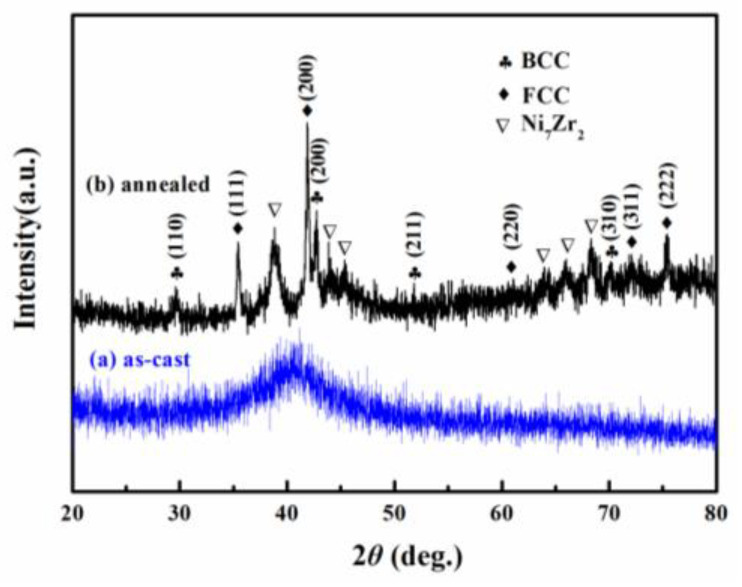
XRD patterns of Ø3 mm Ti_20_Zr_20_Cu_20_Ni_20_Be_20_ rod sample (**a**) as-cast, (**b**) annealed [35].

**Figure 3:**
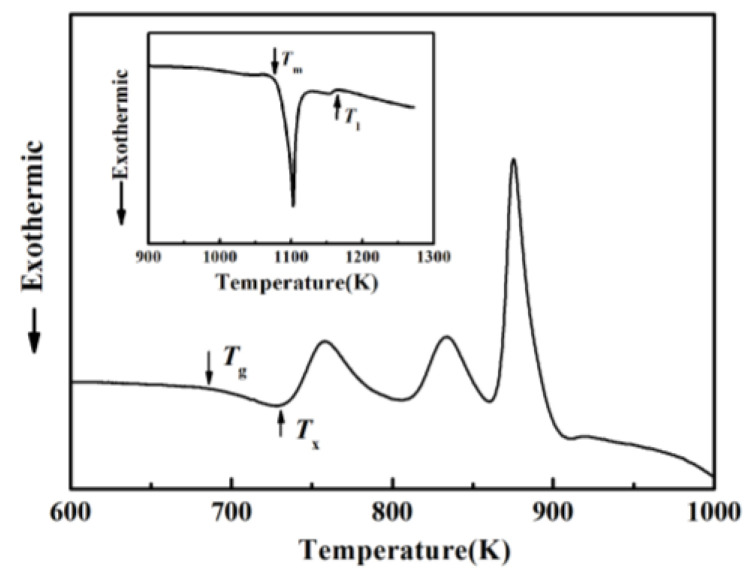
DSC curve of Ø3 mm Ti_20_Zr_20_Cu_20_Ni_20_Be_20_ sample. Inset shows melting behavior [35].

**Figure 4:**
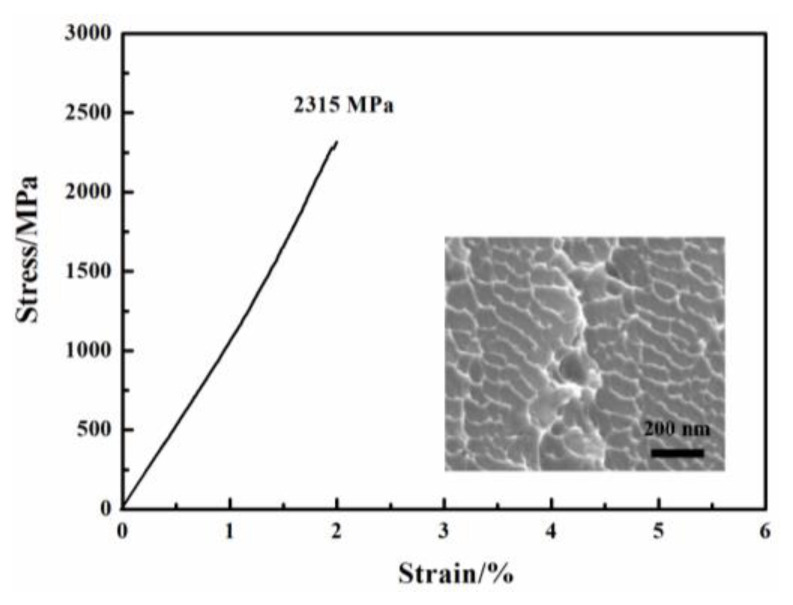
Stress–strain curve of Ø3 mm × 6 mm Ti_20_Zr_20_Cu_20_Ni_20_Be_20_ sample. Inset shows fracture morphology after compression [35].

Figure 1 shows the composition design approach of the Ti_20_Zr_20_Cu_20_Ni_20_Be_20_ HEAA. An equal-atomic Ti_20_Zr_20_Cu_20_Ni_20_Be_20_ high entropy alloy was designed from quinary BMGs with good GFA. The Ø3 mm Ti_20_Zr_20_Cu_20_Ni_20_Be_20_ rod sample was prepared by the copper mold casting technique. Its XRD spectra was shown in Figure 2a. No sharp diffraction peak corresponding to the crystalline phase was observed in the Ø3 mm Ti_20_Zr_20_Cu_20_Ni_20_Be_20_ sample, indicating that this alloy possesses a fully amorphous structure. However, the critical diameter of the Ti_20_Zr_20_Cu_20_Ni_20_Be_20_ BMG sample is only 3 mm, much smaller than that of Zr_41.2_Ti_13.8_Cu_12.5_Ni_10_Be_22.5_ (Vit1) and Ti_32.8_Zr_30.2_Ni_5.3_Cu_9_Be_22.7_ BMGs. The glass transition temperature (*T*_g_), initial crystallization temperature (*T*_x_), melting temperature (*T*_m_), and liquidus temperature (*T*_l_) are marked with arrows in Figure 3. *T*_g_, *T*_x_, *T*_m_, and *T*_l_ are measured to be 683 K, 729 K, 1076 K, and 1161 K, respectively. This high-entropy BMG possesses a high compressive fracture strength of 2315 MPa for Ø3 mm × 6 mm sample, higher than that of Vit1 alloy, which is attributed to high entropy effect as well as high Ni content (Figure 4) [35]. In a uniaxial compressive experiment, it breaks in a brittle manner without plasticity. The present result provides a successful example of HEAA composition design by selecting five elements from quinary BMG with good GFA, despite the fact that the GFA of the designed high-entropy BMG is not large enough. Then, further study for improving the GFA of the high-entropy amorphous alloys is important and necessary.

### 2.2. Designing HEAAs by Similar Element Substitution/Addition

**Figure 5:**
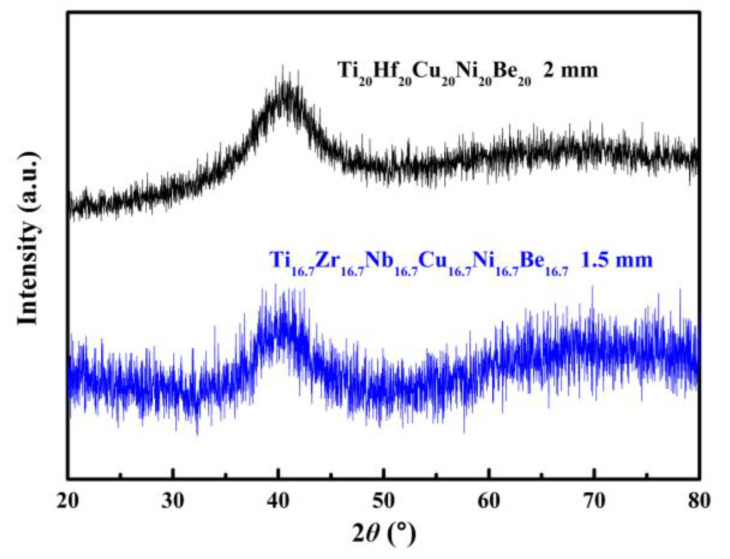
XRD patterns of Ø2 mm Ti_20_Hf_20_Cu_20_Ni_20_Be_20_ and Ø1.5 mm Ti_16.7_Zr_16.7_Nb_16.7_Cu_16.7_Ni_16.7_Be_16.7_ sample [54].

**Figure 6:**
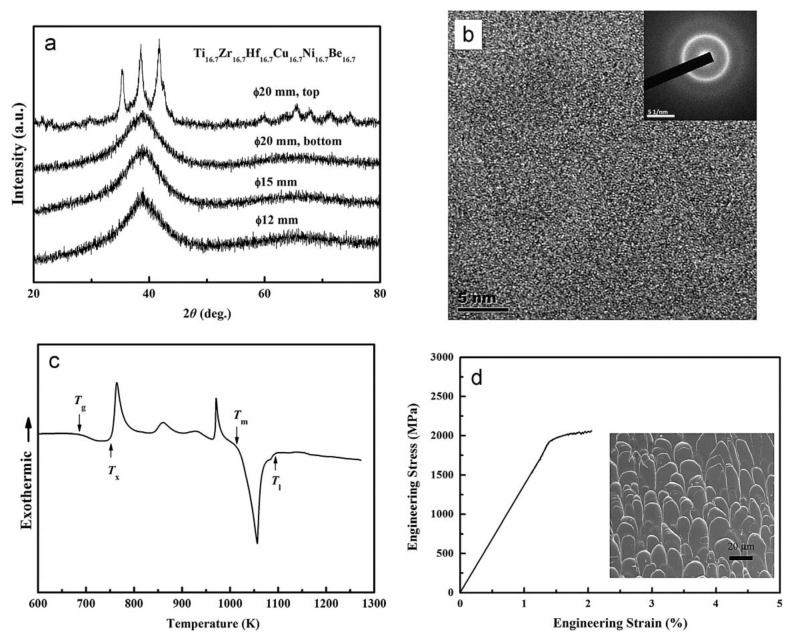
(**a**) XRD spectra of the Ø12 mm, Ø15 mm and Ø20 mm rod samples of Ti_16.7_Zr_16.7_Hf_16.7_Cu_16.7_Ni_16.7_Be_16.7_ alloy. (**b**) The HRTEM image of the Ø15 mm glassy rod (inset: SAED pattern). (**c**) The DSC curve and (**d**) stress–strain curve of a Ø3 mm × 6 mm glassy sample (inset: SEM image) [36].

Similar element substitution/addition was proved to be an effective composition design method in traditional bulk metallic glasses [12,13], so it is reasonable to suppose that it may still work in HEAA. Hf and Zr are members of the same group in the periodic table of elements; they also possess similar atomic radii and chemical properties. Then Hf was used to replace the Zr element in Ti_20_Zr_20_Cu_20_Ni_20_Be_20_ HE-BMG. Therefore, a Ti_20_Hf_20_Cu_20_Ni_20_Be_20_ alloy was designed. It possesses an amorphous structure and its critical diameter is 2 mm, as shown in Figure 5 [54]. Moreover, Nb and Zr are also very close in the periodic table of elements, so Hf and Nb were added to the Ti_20_Zr_20_Cu_20_Ni_20_Be_20_ HE-BMG as a sixth alloying element, respectively. Accordingly, Ti_16.7_Zr_16.7_Nb_16.7_Cu_16.7_Ni_16.7_Be_16.7_ with a critical diameter of 1.5 mm (Figure 5) [54] and Ti_16.7_Zr_16.7_Hf_16.7_Cu_16.7_Ni_16.7_Be_16.7_ with a critical diameter of 15 mm (Figure 6a,b) [36] were designed and developed. Surprisingly, the Ti_16.7_Zr_16.7_Hf_16.7_Cu_16.7_Ni_16.7_Be_16.7_ senary HE-BMG possesses a critical size 10 times that of Ti_16.7_Zr_16.7_Nb_16.7_Cu_16.7_Ni_16.7_Be_16.7_ HE-BMG, and it refreshes our cognition about GFA in an equal-atomic high entropy alloy system. Before this alloy, the largest HE-BMG with an equal-atomic concentration is the Pd_20_Pt_20_Cu_20_Ni_20_P_20_ alloy, and its critical diameter is 10 mm by fluxing method [33]. The composition design approach for the Ti_20_Hf_20_Cu_20_Ni_20_Be_20_ alloy, Ti_16.7_Zr_16.7_Nb_16.7_Cu_16.7_Ni_16.7_Be_16.7_ alloy and Ti_16.7_Zr_16.7_Hf_16.7_Cu_16.7_Ni_16.7_Be_16.7_ alloy is also demonstrated in Figure 1.

**Figure 7:**
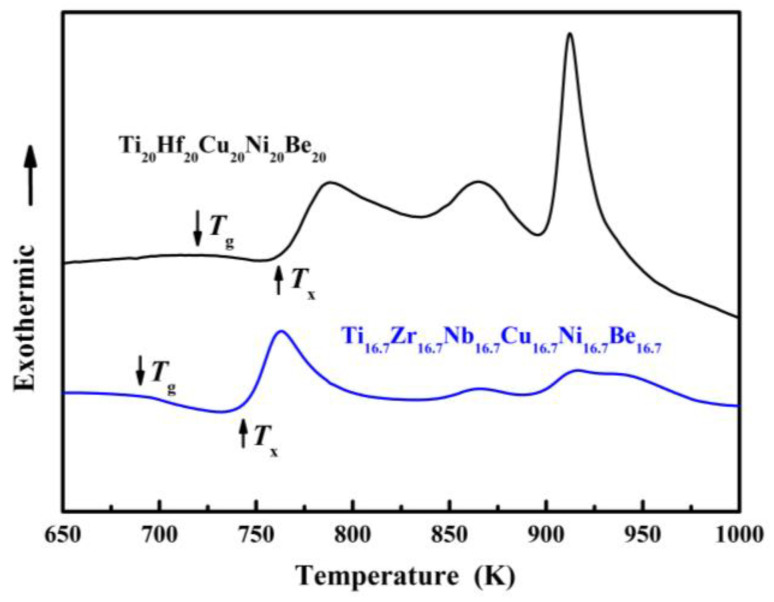
DSC curves of Ø2 mm Ti_20_Hf_20_Cu_20_Ni_20_Be_20_ and Ø1.5 mm Ti_16.7_Zr_16.7_Nb_16.7_Cu_16.7_Ni_16.7_Be_16.7_ sample [54].

**Figure 8:**
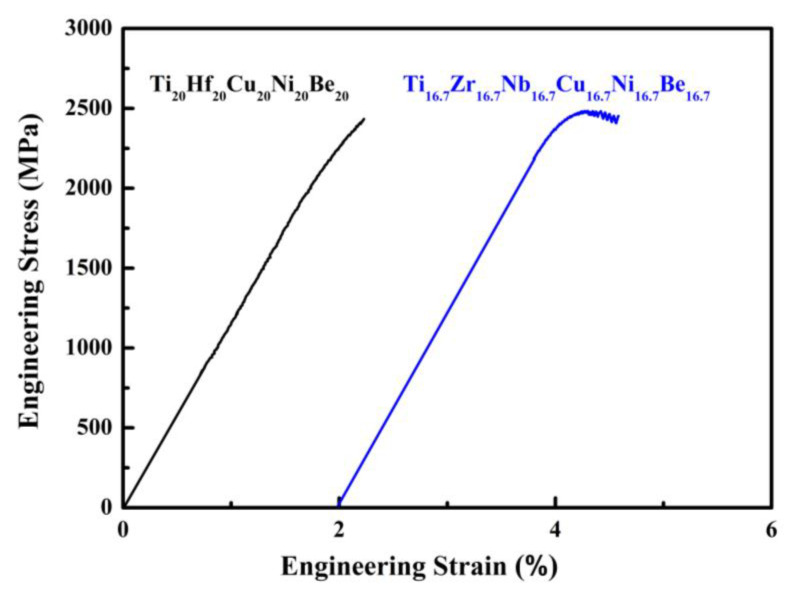
Stress–strain curves of Ø2 mm × 4 mm Ti_20_Hf_20_Cu_20_Ni_20_Be_20_ and Ø1.5 mm × 3 mm Ti_16.7_Zr_16.7_Nb_16.7_Cu_16.7_Ni_16.7_Be_16.7_ sample [54].

For Ti_20_Hf_20_Cu_20_Ni_20_Be_20_ alloy, *T*_g_, *T*_x_, *T*_m_, and *T*_l_ are measured to be 717 K, 760 K, 1095 K, and 1220 K, respectively (Figure 7). Its compressive fracture strength is 2425 MPa for Ø2 mm × 4 mm sample, and it also breaks without plasticity (Figure 8) [54]. For Ti_16.7_Zr_16.7_Nb_16.7_Cu_16.7_Ni_16.7_Be_16.7_ alloy, *T*_g_, *T*_x_, *T*_m_, and *T*_l_ are measured to be 684 K, 739 K, 1066 K, and 1218 K, respectively (Figure 7). Its yield strength, fracture strength, and plasticity are 2330 MPa, 2450 MPa, and 0.5% for the Ø1.5 mm × 3 mm sample, respectively (Figure 8) [54]. For the Ti_16.7_Zr_16.7_Hf_16.7_Cu_16.7_Ni_16.7_Be_16.7_ alloy, *T*_g_, *T*_x_, *T*_m_, and *T*_l_ are measured to be 681 K, 751 K, 1019 K, and 1100 K, respectively (Figure 6c). Its yield strength, fracture strength, and plasticity are 1943 MPa, 2064 MPa, and 0.6% for the Ø3 mm × 6 mm sample, respectively (Figure 6d) [36]. These alloys possess high thermal stability and high strength; the relationship between the high entropy effect and properties will be discussed later.

The fracture surface morphology of Ti_20_Zr_20_Cu_20_Ni_20_Be_20_ was shown as an inset in Figure 4. The nanowave structure is observed on the fracture surface. This is consistent with its brittle failure feature. In contrast, a typical vein pattern has been observed for the Ti_16.7_Zr_16.7_Hf_16.7_Cu_16.7_Ni_16.7_Be_16.7_ alloy (inset in Figure 6d); it also coincides with its plastic deformation behavior. The fracture surface morphology is in agreement with compression experiment results [35,36].

**Figure 9:**
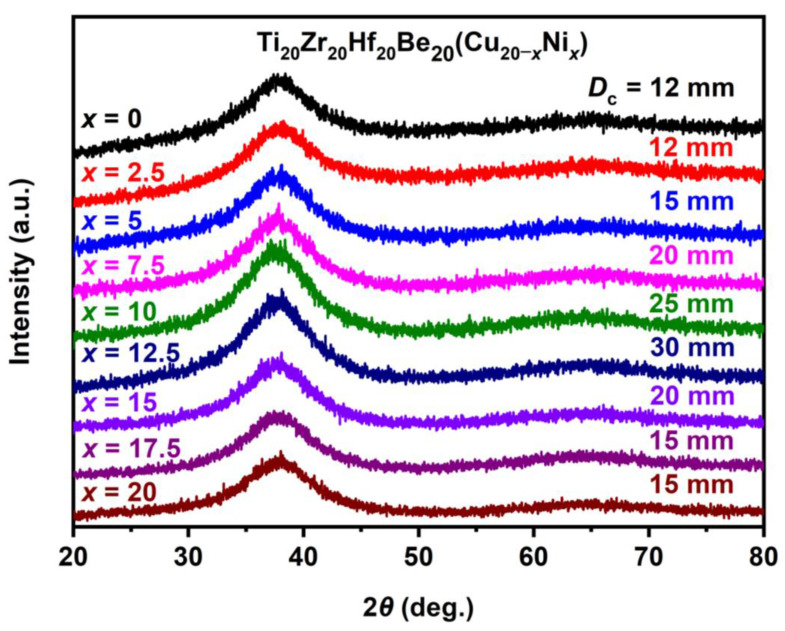
XRD patterns of Ti_20_Zr_20_Hf_20_Be_20_(Cu_20–_*_x_*Ni*_x_*) (*x* = 0, 2.5, 5, 7.5, 10, 12.5, 15, 17.5, 20) HE-BMGs [38].

Stimulated by the Ti_16.7_Zr_16.7_Hf_16.7_Cu_16.7_Ni_16.7_Be_16.7_ alloy with good GFA (critical diameter reaches 15 mm), the Ti–Zr–Hf–Cu–Ni–Be alloys with varied Cu/Ni ratio have been studied since Cu and Ni are also very close in the periodic table of elements, and the atomic radius difference is very small. Experimental results show that a series of Ti_20_Zr_20_Hf_20_Be_20_(Cu(Cu_20–_*_x_*Ni*_x_*) (*x* = 0, 2.5, 5, 7.5, 10, 12.5, 15, 17.5, 20) HE-BMGs with critical diameters of 12–30 mm were designed and developed (Figure 9) [37,38]. The composition design approach was also demonstrated in Figure 1. This series of high-entropy alloys exhibit good glass-forming ability; all of them possess a critical diameter larger than 12 mm and the best glass former, namely the Ti_20_Zr_20_Hf_20_Cu_7.5_Ni_12.5_Be_20_ alloy, reaches a critical diameter of 30 mm, larger than most reported HE-BMGs. It indicates that high entropy alloys can also possess good glass-forming ability.

This progress indicates that similar element substitution/addition is an effective composition design method in exploring HEAAs, just as in traditional BMGs. The present results greatly enlarged the family of HEAAs with high GFA and inspired the researcher’s interest in this field.

### 2.3. Designing HEAAs Based on Existing Ternary/Quaternary Bulk Metallic Glasses

After more than sixty years of research, lots of results were accumulated on bulk metallic glasses, especially on ternary and quaternary amorphous alloys. Naturally, it is supposed to mix five elements or more from these glass-forming alloys together to form a high entropy alloy; maybe it is still very advantageous for amorphous structure formation in terms of dense atomic packing (adequate atomic radius difference) and strong elemental affinity (large negative mixing enthalpy). For example, the critical diameter of Pd_40_Cu_30_Ni_10_P_20_ [5], Pd_42.5_Cu_30_Ni_7.5_P_20_ [16], and Pt_47.5_Cu_27_Ni_9.5_P_21_ [8] alloys are ≥75 mm, 80 mm, and 20 mm, respectively. Accordingly, the Pd_20_Pt_20_Cu_20_Ni_20_P_20_ HE-BMG with a critical diameter of 10 mm [33] can be made.

**Figure 10:**
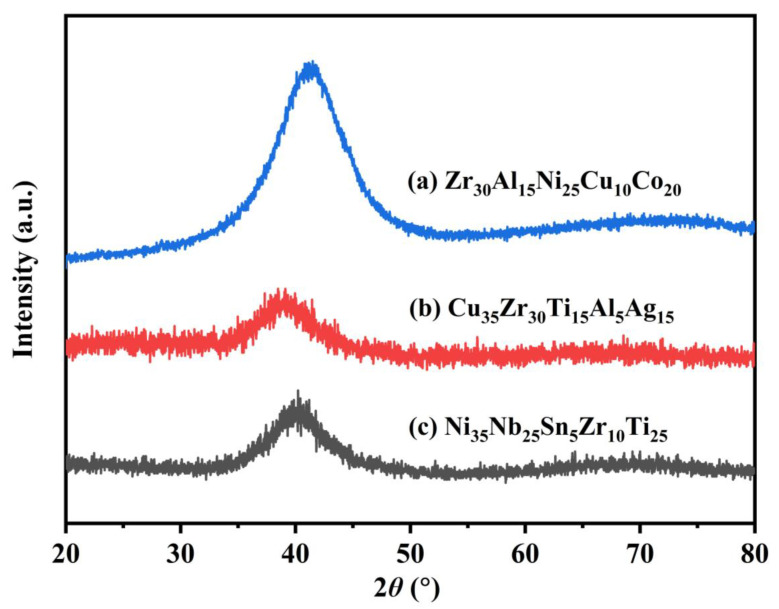
Several HEAA ribbons designed based on ternary bulk metallic glass (**a**) Zr_30_Al_15_Ni_25_Cu_10_Co_20_; (**b**) Cu_35_Zr_30_Ti_15_Al_5_Ag_15_; (**c**) Ni_35_Nb_25_Sn_5_Zr_10_Ti_25_ [55].

Guided by the idea mentioned above, the authors applied for two patents for HEAA composition design, and they were authorized. That is CN112981279B, “Designing quinary high entropy amorphous alloys based on element combinations from three ternary amorphous alloys and its preparation method” [55], and CN112466409B, “Composition design method for quinary high entropy amorphous alloys based on element combinations from two quaternary amorphous alloys” [61], respectively. The main procedure can be divided into three steps. (1) Find out several alloy compositions with good glass-forming ability reported in literature; (2) Select elements from these alloys to form quinary high entropy alloy; (3) Verify the structure of the newly developed alloy through experiments. It may possess an amorphous structure with high probability, at least in a ribbon form, prepared by the melt-spinning method. Some of them may also form bulk metallic glasses by copper mold casting. For example, based on Zr_60_Al_20_Ni_20_ [2], Zr_65_Al_7.5_Cu_27.5_ [3] and Zr_53_Al_23.5_Co_23.5_ [9] amorphous alloys, a Zr_30_Al_15_Ni_25_Cu_10_Co_20_ HEAA ribbon was designed and fabricated (Figure 10a). Based on Cu_60_Zr_30_Ti_10_ [6], Cu_49_Zr_45_Al_6_ [10], and Cu_54_Zr_36_Ag_10_ [11] alloys, a Cu_35_Zr_30_Ti_15_Al_5_Ag_15_ HEAA was designed (Figure 10b). Based on Ni_60_Nb_35_Sn_5_ [7], Ni_50_Nb_28_Zr_22_ [14], and Ni_60_Nb_25_Ti_15_ [20] alloys, a Ni_35_Nb_25_Sn_5_Zr_10_Ti_25_ HEAA was obtained (Figure 10c) [55]. Quinary HEAAs can be designed from two kinds of quaternary amorphous alloys in a similar way [61]. The current method is based on existing experimental results, and it conforms to theoretical analysis; multiple high entropy amorphous alloy components can be developed quickly. In this way, it can reduce the workload of trial and error, resulting in high composition design efficiency.

## 3. Correlation between Entropy and Property of High Entropy Amorphous Alloys

The most prominent feature of HEAA, as compared with traditional alloys, lies in its high entropy. Then, the correlation between entropy and the properties of high-entropy amorphous alloys becomes an interesting topic. For comparison, the thermal property (as indicated by *T*_g_, *T*_x_, *T*_m_, and *T*_l_), mechanical property (as indicated by fracture strength *σ*_max_), glass-forming ability (as indicated by critical diameter *D*_c_), and mixing entropy (Δ*S*_mix_) of five typical alloys were listed in Table 1. It is very clear that the mixing entropy of the Zr_41.2_Ti_13.8_Cu_12.5_Ni_10_Be_22.5_ (Vit1) [4] and Ti_32.8_Zr_30.2_Ni_5.3_Cu_9_Be_22.7_ alloys [15] is relatively lower compared with Ti_20_Zr_20_Cu_20_Ni_20_Be_20_, Ti_16.7_Zr_16.7_Hf_16.7_Cu_16.7_Ni_16.7_Be_16.7_ and Ti_20_Zr_20_Hf_20_Cu_7.5_Ni_12.5_Be_20_ alloys. In contrast, the former two alloys (No. 1–No. 2) possess lower thermal stability (smaller *T*_g_, *T*_l_) and smaller fracture strength *σ*_max_ than the latter three alloys (No. 3–No. 5) in general. In fact, Zr_41.2_Ti_13.8_Cu_12.5_Ni_10_Be_22.5_ (Vit1) and Ti_32.8_Zr_30.2_Ni_5.3_Cu_9_Be_22.7_ alloys can also be classified as HEAAs in a broad sense, while the mixing entropy is slightly lower than equal-atomic or near equal-atomic alloys. Higher mixing entropy leads to larger lattice distortion and sluggish atomic diffusion; consequently it obtained stronger ability against thermal/mechanical load. Therefore, high entropy exerts a positive effect on the thermal stability and mechanical property of HEAAs.

The factors influencing GFA are very complicated. On the one hand, as entropy increased, the melt tends to be very stable especially under high temperature, which is beneficial for glass formation (see comparison of Ti_16.7_Zr_16.7_Hf_16.7_Cu_16.7_Ni_16.7_Be_16.7_ and Ti_20_Zr_20_Cu_20_Ni_20_Be_20_ in Table 1). On the other hand, the higher liquidus temperature is harmful to glass formation. Overall, the glass-forming ability of the latter three alloys (No. 3–No. 5) in Table 1 possess poorer glass-forming ability (smaller *D*_c_) than the former two alloys (No. 1–No. 2). Reducing the melting point of an alloy (closer to the eutectic composition point) may be beneficial for amorphous formation. The liquidus temperature of No. 1–No. 2 is lower than No. 3–No. 5 alloys in Table 1; they possess larger GFA despite their lower mixing entropy. This can also be verified in the Ti_20_Zr_20_Hf_20_Be_20_Cu_20−x_Ni_x_ (x = 0–20) alloy system [37,38]. That is to say, the Ti_20_Zr_20_Hf_20_Cu_7.5_Ni_12.5_Be_20_ sample demonstrates the lowest liquidus temperature (1040 K) while the largest critical diameter (30 mm) in the Ti_20_Zr_20_Hf_20_Be_20_Cu_20−x_Ni_x_ (x = 0–20) alloy system [37,38]. Its entropy is very high, but not the highest compared with Ti_20_Zr_20_Hf_20_Cu_10_Ni_10_Be_20_ [38] and Ti_16.7_Zr_16.7_Hf_16.7_Cu_16.7_Ni_16.7_Be_16.7_ alloys [36]. The glass-forming ability is somewhat a competition between the high entropy effect and eutectic point effect in the current Ti–Zr–Hf–Cu–Ni–Be alloy system. However, the amorphous formation mechanism needs more in-depth investigation.

## 4. Potential Composition Design Method for HEAAs

HEAA is an intersection of amorphous alloys and high entropy alloys. Therefore, research results on the theory of amorphous formation and phase formation rules of high entropy alloy phases can provide useful inspiration. For example, Takeuchi et al. proposed that the composition–configurational entropy (C–CE) diagram is helpful in designing Pd_20_Pt_20_(TM1)_20_(TM2)_20_P_20_ alloys (TM1, TM2 = Fe, Co, Ni, Cu) [63]. They also pointed out that *S*_σ_/*k*_B_ − Δ*H*_mix_ and a phase diagram can play an important role in HEAA composition design [64]. Moreover, Li et al. proposed a simplified combinatorial approach to design high-strength, high-temperature Ir–Ni–Ta–(B) bulk metallic glass; the key points lie in the relationship between glass-forming ability and electrical resistivity. By high-throughput methods, the efficiency of the experiment was enhanced greatly [65]. Wu et al. report a rapid design of superior high-entropy alloys based on existing eutectic high-entropy alloys [66]. Eutectic point criteria are very important for amorphous alloy formation. These research progress in amorphous alloys and high entropy alloys will play an important role in the future development of HEAAs.

Although several methods have been mentioned above, most of them are still based on individual experience and trial-and-error methods. It is very time-consuming. In recent years, with the development of big data and its close integration with various disciplines, the application of artificial intelligence technology has become increasingly common. Machine learning (ML) especially was applied in the development of amorphous alloys and high entropy alloys. For example, Ren et al. trained an ML model to find new metallic glass in the Co–V–Zr alloys; accuracy was improved after refinement, and it can provide guidance to the rapid discovery of three new glass-forming systems [67]. Huang et al. employed ML algorithms to explore phase selection rules efficiently; it was found that the trained ANN model performs better than SVM and KNN in accuracy [68]. Mastropietro et al. used multiple linear regression and tree boosting to predict the maximum amorphous diameter of Fe-based BMGs; the R^2^ value was increased from 0.71 to 0.90 after training [69]. Reddy et al. predicted the glass-forming ability of a BMG by ML based on elemental composition alone [70]. Schultz et al. tried to link characteristic temperature and glass-forming ability in BMGs by ML; it was found that the critical cooling rate (*R*_c_) might be a better target for machine learning model prediction than critical casting diameter (*D*_c_) [71]. Rao et al. identified two high-entropy Invar alloys with extremely low thermal expansion coefficients via ML [72]. Vazquez et al. assessed the elastic properties of Nb–Ta–Mo–W–V-based HEAs via descriptor-based ML framework models [73]. Wieczerzak et al. investigated the mechanical properties of the CuAgZr metallic glass system assisted by ML. It was found that leveraging the fine-tuned MLP algorithm enabled the prediction of the hardness of untested alloys in the virtual space, and can serve as a valuable guide for further exploration [74]. Dewangan et al. presented a review of applications of artificial neural network (ANN) modeling in predicting phase formation, microstructures, and mechanical properties of HEAs [75]. In general, intelligent technologies represented by machine learning may promote the development of HEAAs in the near future.

## 5. Potential Applications for HEAAs in Future

The ultimate goal of developing new materials is to search for industrial applications and promote social development. Due to their complicated composition and structural characteristics, high entropy amorphous alloys exhibit a series of unique physical, chemical, and mechanical properties, and they may be applied in many fields.

**Figure 11:**
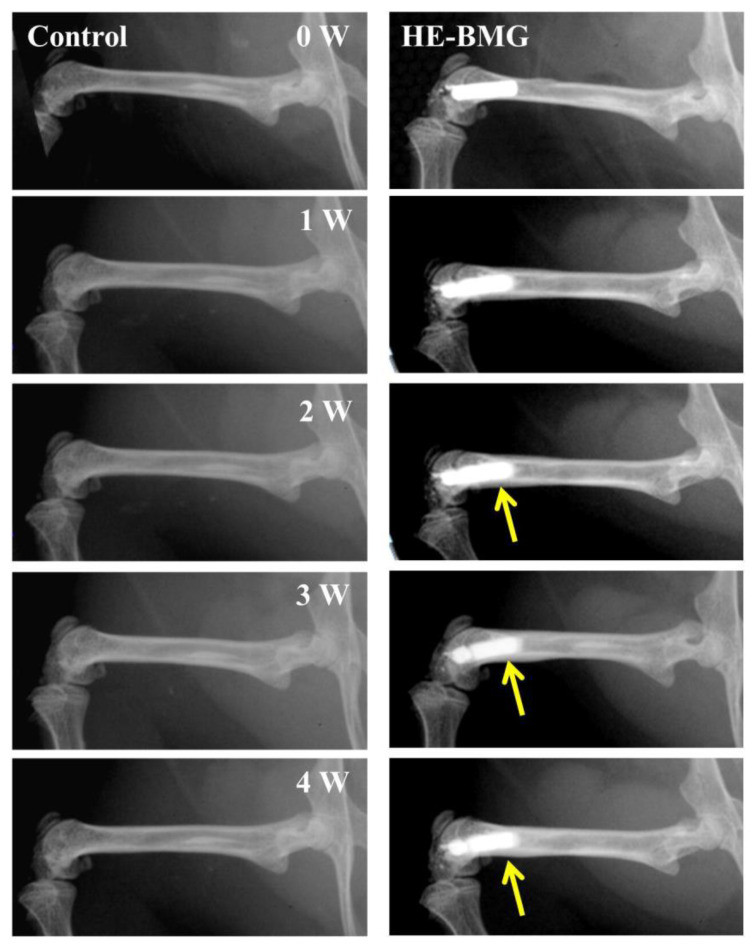
Ca_20_Mg_20_Zn_20_Sr_20_Yb_20_ HEAA as a biomaterial for orthopedic applications, yellow arrows indicated formation of new bone [34].

Biomedical application. For example, the Ca_20_Mg_20_Zn_20_Sr_20_Yb_20_ HEAA as a biomaterial for orthopedic applications was investigated in both in vitro and in vivo environments. Results showed that it could stimulate the proliferation and differentiation of cultured osteoblasts. Moreover, they did not show any obvious degradation after 4 weeks of implantation, they can promote osteogenesis and new bone formation after 2 weeks of implantation (Figure 11) [34]. The Ti–Zr–Nb–Hf–Si HEAA possesses high thermal stability and excellent corrosion properties in simulated body fluid. Moreover, the weak paramagnetic nature and superior radiopacity offer compatibility with medical diagnostic imaging systems [48]. The NbTaTiVZr(O) HEAA was also reported to possess enhanced surface protection and superior biocompatibility [56]. It means that HEAA may become a potential candidate for biomedical applications.

**Figure 12:**
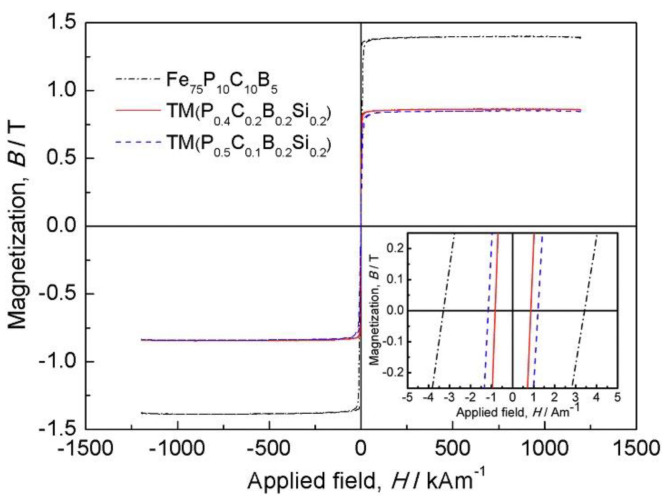
Fe_25_Co_25_Ni_25_(P, C, B, Si)_25_ HEAA as soft magnetic material [40].

Ferromagnetic application. For example, Fe_25_Co_25_Ni_25_(P, C, B, Si)_25_ alloys possess high strength (~3000 MPa), high saturation magnetization (>0.80 T), low coercive force (~1 A/m), and high effective permeability (Figure 12) [40]. They may be applied as soft magnetic materials.

**Figure 13:**
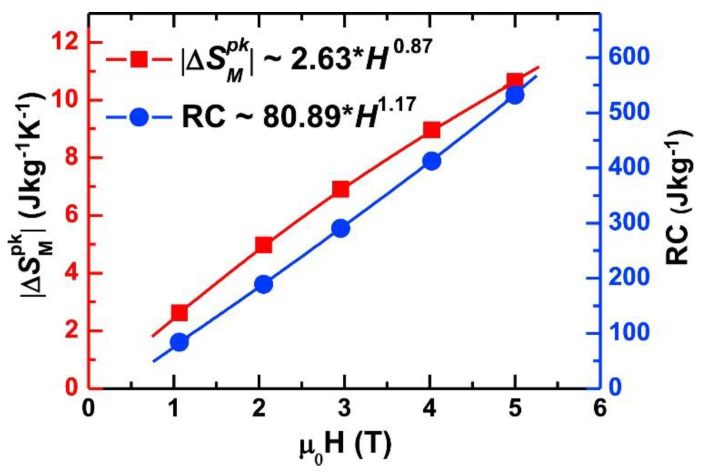
Gd_10_Tb_10_Dy_10_Ho_10_Er_10_Y_10_Ni_10_Co_10_Ag_10_Al_10_ HEAA as a magnetic refrigerant [41].

Magnetic refrigerant. For example, Gd_10_Tb_10_Dy_10_Ho_10_Er_10_Y_10_Ni_10_Co_10_Ag_10_Al_10_ HEAA showed large magnetic entropy changes as the temperature changed. The reason can be attributed to a combination of spin glass behavior and complicated compositions. Moreover, the magnetocaloric properties of HEAAs can be easily adjusted by changing elements or configurational entropy (Figure 13) [41]. The large refrigerant capacity means that HEAAs are promising candidate materials for use as magnetic refrigerants.

**Figure 14:**
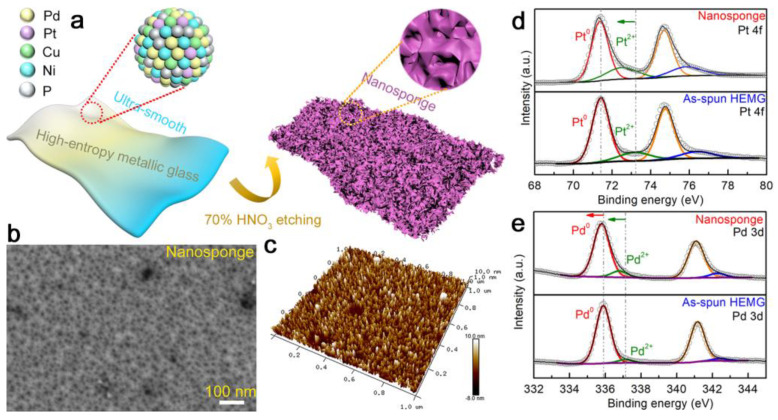
PdPtCuNiP HEAA as hydrogen evolution reaction catalytic material [51].

Catalytic performance. For example, PdPtCuNiP high entropy metallic glass ribbon with a nanosponge-like surface morphology displays outstanding hydrogen evolution reaction activity in both alkaline and acidic conditions, outperforming most currently available electro-catalysts (Figure 14). Moreover, the process is very stable even after 100 h, indicating great potential for commercialization [51].

**Figure 15:**
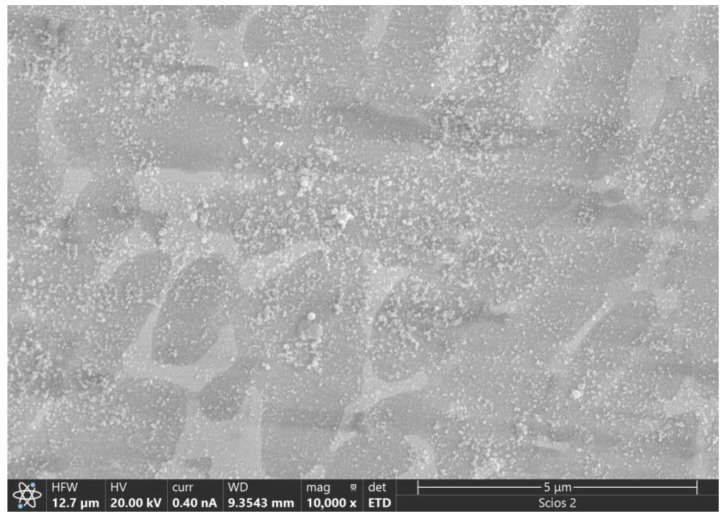
Microstructure of Ti–Zr–Cu–Ni–Al–Co high entropy amorphous/nanocrystalline coating [60].

Wear-resistant material. For example, Ti–Zr–Cu–Ni–Al–Co high entropy amorphous/nanocrystalline coatings processed by laser cladding possess characteristics of high hardness, fine microstructure, and good wear resistance. Its microstructure is demonstrated in Figure 15. The Vickers hardness exceeds 790 HV, and its wear loss amount was reduced to half of the TC4 matrix, demonstrating excellent wear resistance properties. It indicates that HEAA may be a suitable material for wear-resistant applications [60].

## 6. Summary

In general, the high entropy amorphous alloy (HEAA) is a kind of material with a special composition and microstructure. After the researchers’ efforts in recent years, many achievements have been made, while more work is still needed in the future.

(1) Directly adjusting the atomic ratio from existing quinary bulk metallic glass, similar element addition/substitution, and combining elements from existing ternary/quaternary alloys with good glass-forming ability was proved to be an effective method for designing HEAAs; it provides possibilities for utilizing existing research data to develop more new HEAAs.
(2) The glass-forming ability of HEAAs was affected by many factors; both the high-entropy effect and eutectic point criteria could impose positive influences.
(3) Due to their unique properties, HEAAs possess potential applications as biomedical material, magnetic refrigerants, ferromagnetic material, catalytic material, wear-resistant material, and other uses.

## Figures and Tables

**Table 1:** Thermal and mechanical properties of several Ti–Zr–(Hf)–Cu–Ni–Be HEAAs.

Alloy No.	Composition	*T*_g_ (K)	*T*_x_ (K)	*T*_m_ (K)	*T*_l_ (K)	*σ*_max_ (MPa)	*D*_c_ (mm)	Δ*S*_mix_ (J/(mol·K))	Year
1	Zr_41.2_Ti_13.8_Cu_12.5_Ni_10_Be_22.5_ (Vit1)	625	705	937	993	(<2000)	>50	12.17	1993 [4]
2	Ti_32.8_Zr_30.2_Ni_5.3_Cu_9_Be_22.7_	611	655	-	961	1831	>50	11.94	2010 [15]
3	Ti_20_Zr_20_Cu_20_Ni_20_Be_20_	683	729	1076	1161	2315	3	13.38	2013 [35]
4	Ti_16.7_Zr_16.7_Hf_16.7_Cu_16.7_Ni_16.7_Be_16.7_	681	751	1019	1100	2064	15	14.90	2014 [36]
5	Ti_20_Zr_20_Hf_20_Cu_7.5_Ni_12.5_Be_20_	632	684	951	1040	2124	30	14.53	2015 [38]
